# HEALTHCARE LITERACY AMONG INFORMAL CAREGIVERS: THE ROLE OF SOCIAL SUPPORT SYSTEM AND BURNOUT

**DOI:** 10.1093/geroni/igac059.3118

**Published:** 2022-12-20

**Authors:** Robyn Medcalf, Jessika Dayrit, Shirley Salom-Bail, Cesz Islaya, Shelly Foutz-Lowe, Renuka Alladi

**Affiliations:** Department of Veterans Affairs, Palo Alto Health Care System, Palo Alto, California, United States; Washington University in St. Louis, St. Louis, Missouri, United States; Department of Veterans Affairs, Palo Alto Health Care System, Palo Alto, California, United States; Department of Veterans Affairs, Palo Alto Health Care System, Palo Alto, California, United States; Department of Veterans Affairs, Palo Alto Health Care System, Palo Alto, California, United States; Department of Veterans Affairs, Palo Alto Health Care System, Palo Alto, California, United States

## Abstract

Informal caregivers are integral components in the lives of individuals with health problems. However, little is known if the number of hours of informal care correlates with healthcare literacy levels. Thus, using the Center for Disease Control (CDC) Behavioral Risk Factor Surveillance (BRFSS) 2016 public dataset, the aim of this study examines the association between informal caregivers’ hours of care and their health literacy level, by controlling their social/emotional support, type of care they provide, and socioeconomic level. Data was weighted for complex sampling design and logistic regression to examine the data (Nf3,249). The study’s results show that being full-time or part-time caregiver has no association to healthcare literacy in verbal (OR=0.99, 95% CI=0.57–1.71) and written (OR=1.21, 95% CI=0.78–1.85) comprehensions. Further, caregiver’s type of care does not correlate to healthcare literacy level in verbal (OR=0.87, 95% CI=0.58–1.32; OR=0.93, 95% CI=0.59–1.45) and written (OR=0.91, 95% CI=0.66–1.27; OR=0.81, 95% CI=0.57–1.16) comprehensions. However, caregivers who rarely-to-never receive social/emotional support are four times more likely to have difficulties understanding verbal health information than those who have always-to-usually receive social/emotional support (OR=4.02, 95% CI=2.45–6.61). Caregivers who rarely-to-never receive social/emotional support are two times more likely to have difficulties understanding written health information compared to those that have always-to-usually receive social/emotional support (OR=2.30, 95% CI=1.51–3.51). Caregivers with lower socioeconomic backgrounds are at higher probability of low healthcare literacy level. Our study suggests further longitudinal and qualitative research, to understand healthcare literacy level among informal caregivers, and how social support systems correlates to healthcare literacy level.

